# Systems biology and machine learning approaches identify drug targets in diabetic nephropathy

**DOI:** 10.1038/s41598-021-02282-3

**Published:** 2021-12-06

**Authors:** Maryam Abedi, Hamid Reza Marateb, Mohammad Reza Mohebian, Seyed Hamid Aghaee-Bakhtiari, Seyed Mahdi Nassiri, Yousof Gheisari

**Affiliations:** 1 Regenerative Medicine Research Center, Isfahan University of Medical Sciences, Isfahan, Iran; 2 Biomedical Engineering Department, Engineering Faculty, University of Isfahan, Isfahan, Iran; 3 Department of Automatic Control, Biomedical Engineering Research Center, Universitat Politècnica de Catalunya, BarcelonaTech (UPC), Barcelona, Spain; 4 Department of Electrical and Computer Engineering, University of Saskatchewan, Saskatoon, Canada; 5 Bioinformatics Research Group, Mashhad University of Medical Sciences, Mashhad, Iran; 6 Department of Medical Biotechnology and Nanotechnology, Faculty of Medicine, Mashhad University of Medical Sciences, Mashhad, Iran; 7 Department of Clinical Pathology, Faculty of Veterinary Medicine, University of Tehran, Tehran, Iran; 8 Department of Genetics and Molecular Biology, Isfahan University of Medical Sciences, Isfahan, Iran

**Keywords:** Machine learning, Diabetes complications

## Abstract

Diabetic nephropathy (DN), the leading cause of end-stage renal disease, has become a massive global health burden. Despite considerable efforts, the underlying mechanisms have not yet been comprehensively understood. In this study, a systematic approach was utilized to identify the microRNA signature in DN and to introduce novel drug targets (DTs) in DN. Using microarray profiling followed by qPCR confirmation, 13 and 6 differentially expressed (DE) microRNAs were identified in the kidney cortex and medulla, respectively. The microRNA-target interaction networks for each anatomical compartment were constructed and central nodes were identified. Moreover, enrichment analysis was performed to identify key signaling pathways. To develop a strategy for DT prediction, the human proteome was annotated with 65 biochemical characteristics and 23 network topology parameters. Furthermore, all proteins targeted by at least one FDA-approved drug were identified. Next*, mGMDH-AFS*, a high-performance machine learning algorithm capable of tolerating massive imbalanced size of the classes, was developed to classify DT and non-DT proteins. The sensitivity, specificity, accuracy, and precision of the proposed method were 90%, 86%, 88%, and 89%, respectively. Moreover, it significantly outperformed the state-of-the-art (*P*-value ≤ 0.05) and showed very good diagnostic accuracy and high agreement between predicted and observed class labels. The cortex and medulla networks were then analyzed with this validated machine to identify potential DTs. Among the high-rank DT candidates are Egfr, Prkce, clic5, Kit, and Agtr1a which is a current well-known target in DN. In conclusion, a combination of experimental and computational approaches was exploited to provide a holistic insight into the disorder for introducing novel therapeutic targets.

## Introduction

Diabetic nephropathy (DN) is a major complication in diabetes mellitus and the leading cause of end-stage renal disease (ESRD). Despite the beneficial effects of current drugs such as angiotensin-converting enzyme inhibitors and angiotensin II receptor blockers, DN patients are still reaching ESRD^1^. Therefore, it is critical to understand the molecular mechanisms of this disorder to develop more efficient therapeutic approaches. Systems biology with its quantitative and predictive viewpoints has provided a unique opportunity to explore the complex biological processes involved in the pathogenesis of chronic disorders^2,3^. It allows the generation of holistic maps of interactions between a variety of biomolecules involved in these processes. Considering the pivotal role of microRNAs (miRNAs) in the regulation of a bundle of functionally related genes^4,5^, we were motivated to study the network of DN-associated miRNAs with their targets. Although the role of individual miRNAs in DN has been previously explored^6^, holistic evaluations have just been started^7^.

Valuable insights into the molecular mechanisms of complex disorders have been introduced using systems biology strategies, however, little progress has been made in the translation of this knowledge to the bedside. Although omics technologies, in line with advanced computational techniques, allowed the identification of lots of biomolecules with complex interactions in disease pathogenesis, the identification of appropriate therapeutic targets has remained elusive. To address this challenge, some investigators have proposed that the success of current FDA-approved drugs compared to many ingredients that failed during preclinical and clinical screenings can, at least partly, be attributed to the characteristics of their target proteins^8^. Hence, these potential discriminating properties can be identified and exploited to predict novel drug targets (DT). Based on this assumption, several classic machine learning algorithms have been utilized for feature selection and DT prediction^9–15^. However, these studies suffer from several limitations such as ignoring the unequal frequency of DT and non-DT proteins, inappropriate machine performance measures, or unspecified details of utilized methods. We have here developed a next-generation machine learning method that considers high-level feature interactions and unbalanced DT/non-DT classes.

**Figure 1:**
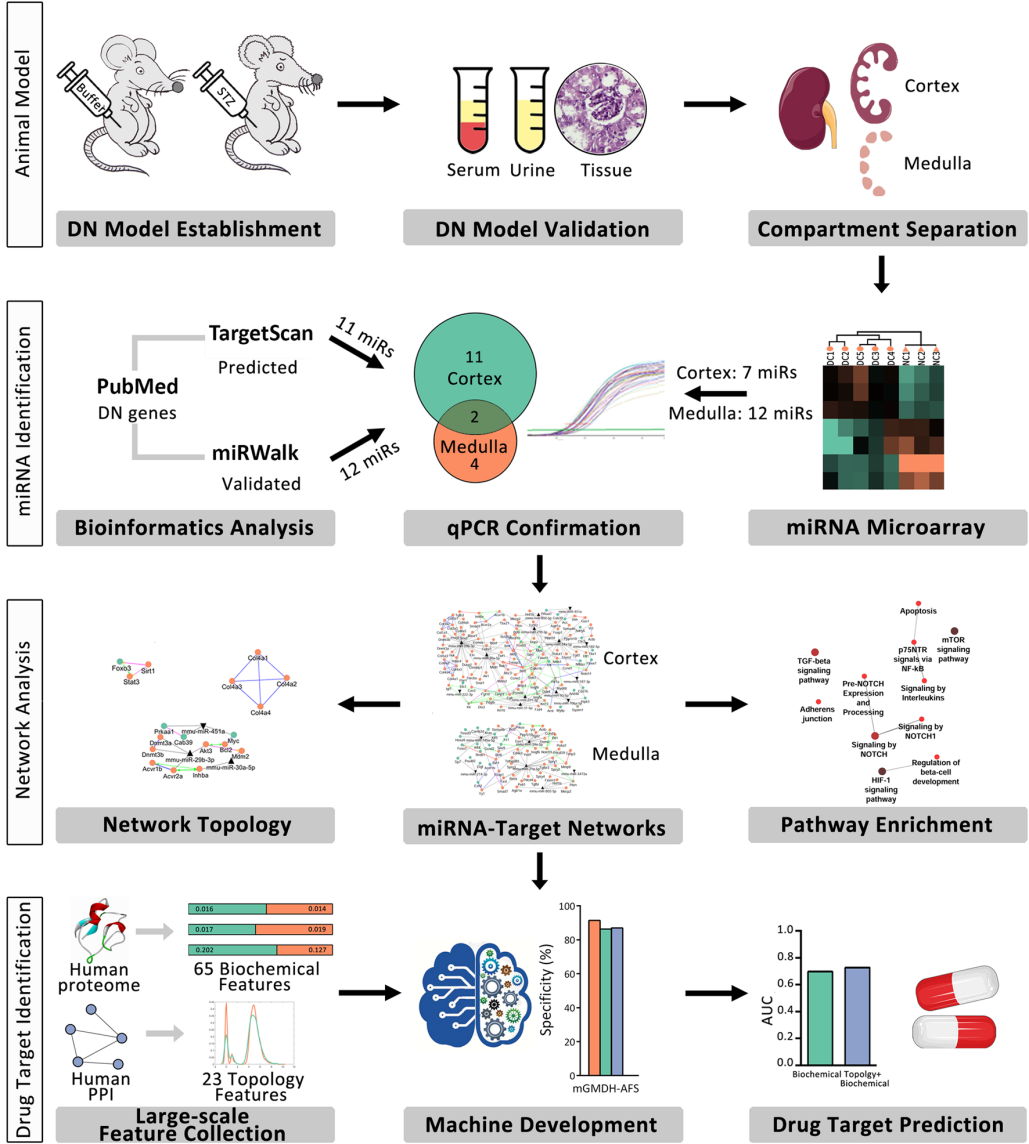
A schematic representation of the study design. This study was aimed at predicting novel drug targets for DN based on the holistic molecular pathogenesis map. Using different experimental and computational methods, the central nodes, key interactions, and signaling pathways of DN were identified. To translate the findings to clinical application, a high-performance machine learning framework, *mGMDH-AFS,* was developed and validated to predict drug targets for all human proteins. This classifier was then applied to candidate novel therapeutic targets in the constructed holistic map of DN. miRs: microRNAs; PPI: protein–protein interaction network.

This study aimed to predict novel targets for DN based on the holistic map of molecular pathogenesis. Several in silico and wet lab steps were pursued to identify the miRNA profile of the disorder and detect novel differentially expressed (DE) miRNAs in the cortex and medulla of diabetic kidneys. Moreover, miRNA-target interaction networks were inferred to identify central nodes and critical interactions. Pathway enrichment analysis also allowed the prediction of affected signaling pathways in this disorder. Next, to translate the findings of this study to clinical application, a high-performance machine learning framework, named "modified Group Method of Data Handling with Automatic Feature Selection (mGMDH-AFS)", was developed and validated for the prediction of DT in human proteome based on a variety of biochemical and network topology features. This classifier was then applied to candidate novel therapeutic targets in the constructed holistic map of DN. The design of this study is schematically presented in Fig. 1.

## Materials and methods

### Diabetic nephropathy mouse model

Male DBA/2 J mice, aged 6–10 weeks, were supplied from the Pasteur Institute of Iran (Tehran, Iran). All animal studies were performed according to the NIH guide for the care and use of laboratory animals^16^. In addition, the study was conducted in compliance with the ARRIVE guidelines. All protocols were approved by the Isfahan University of Medical Sciences Ethics Committee (IR.MUI.MED.REC.1399.933). For five consecutive days, streptozotocin (STZ) was dissolved in sodium citrate buffer and intraperitoneally administered to pre-starved mice at the dose of 40 mg/kg (total amount: 200 mg/kg). Control mice received citrate buffer. Mice were supplied with 10% sucrose water during STZ injection and a few days after to avoid sudden hypoglycemia. As the mice with a lesser extent of diabetes usually do not show renal injury, one week after the final STZ injection, their non-fasting blood glucose was evaluated and those with blood glucose levels below 280 mg/dL were excluded from the study. Three months after the last dose of STZ, mice with the blood glucose range of 300–600 mg/dL were transferred to metabolic cages to collect 24-h urine. Urine albumin concentration was measured with an ELISA test (Exocell, Philadelphia, PA), and urine volume was used to calculate total albumin excretion. Urine-specific gravity, kidney weight, serum glucose, HbA1C, serum creatinine, and urea were also measured. Moreover, kidney tissues were sampled. Histopathologic parameters such as glomerular basement membrane thickening, mesangial matrix, increased mesangial cell proliferation, and diffuse mesangial sclerosis were assessed after Hematoxylin and Eosin (H&E) and Periodic Acid–Schiff (PAS) staining. For each pathology field, 20 serial glomeruli were evaluated, and the percentages of affected glomeruli were calculated. The Mann–Whitney U test was applied for statistical analysis.

### miRNA microarray

The left kidneys were harvested, and the cortex and medulla were separated. The tissues were kept in RNAlater (Qiagen, Valencia, CA, USA) and stored at −70 °C until RNA extraction. The tissues were lysed in 1 mL QIAzol (Qiagen, Valencia, CA, USA) and homogenized with TissueLyser LT (Qiagen). Next, 250 µL chloroform (Merck, Darmstadt, Germany) was added and centrifuged at 12,000 rpm for 20 min at 4 °C after 15 min of incubation. An equal volume of cold ethanol (Merck) was added to the upper aqueous phase in a new tube and incubated at −20 °C overnight after which, the samples were centrifuged at 14,000 g for 45 min at 4 °C. To dislodge the pellet, 75% ethanol was added and centrifuged at 12,000 g for 15 min at 4 °C. The dried pellet was dissolved in double-distilled water. Using a BioPhotometer (Eppendorf, Hamburg, Germany), RNA concentration was measured at 260 nm.

miRNA profiling was performed for the cortex and medulla of five DN and three control mice (total 16 samples). The quality of the microarray experiment was evaluated by the principal component analysis (PCA) and the hierarchical clustering. For hierarchical clustering, the correlation coefficient, and the average linkage methods, as the distance metric, were applied and the heat maps were plotted by ClusterMaker application^17^ of Cytoscape software version 3.2.0^18^. PCA was carried out with ggplot package^19^ of R^20^. The miRNAs with logarithm to base two of fold change (log_2_FC) ≥ 0.5 or ≤ −0.5 were selected.

### Quantitative PCR

Primers were designed with Gene Runner version 3.05 (Hastings Software Inc., Hastings, NY, USA) and Oligo version 7 (Molecular Biology Insights, Inc, USA) software. cDNA was synthesized using RevertAid First-Strand cDNA Synthesis Kit (Thermo Scientific, Vilnius, Lithuania) and PCR thermal cycler machine (Takara Bio, Shiga, Japan). The RNA samples, stem-loop specific primer, and double-distilled water were mixed and incubated at 75 °C for 5 min. The vials were immediately placed on ice, and ten mM dNTP mix, 5X reaction buffer, M-MuLV RT enzyme, and double-distilled water were added and spun briefly. Next, they were incubated at 42 °C for 60 min. The reaction was terminated by heating at 70 °C for 5 min.

QuantiFast SYBR Green PCR kit (Takara Bio) and Rotor-Gene 6000 real-time PCR machine (Corbett, Sydney, Australia) were employed to assess the expression levels of 37 miRNAs as well as Snord70 and Snord68, as internal controls in the same samples used for the microarray experiment. The temperature profile consisted of an initial step of 95 °C for 10 min, followed by 40 cycles of 95 °C for 15 s and 60 °C for 1 min. The data were analyzed using REST 2009 software^21^.

### MicroRNA target identification

Experimentally validated targets of DE miRNAs were collected using miRTarBase database^22^. Targets that were validated by strong evidence were selected. The validated targets were obtained from studies on murine samples for all miRNAs, except for mmu-miR-802-5p and mmu-miR-187-3p that did not have any mouse validated targets; thus, human targets with strong evidence were obtained. A list of genes with potential roles in DN was manually collected from the literature. TargetScan 6.2^23^ and miRWalk databases^24^ were used for predicted, and validated miRNAs targeting these genes, respectively. The miRNAs that were broadly conserved among vertebrates and mammals were selected in TargetScan.

### Network construction and analysis

Using CluePedia plugin version 2.1.7^25^ of Cytoscape software, interaction networks for validated targets of DE miRNAs in the cortex and medulla were constructed by STRING resource^26^. Confidence cut-off was set at 0.8; then, DE miRNAs were merged to the networks. The edges including activation, post-translational modification, binding, and inhibition were allowed to be shown. The topology of the networks was analyzed by NetworkAnalyzer^27^, CytoNCA^28^, and CentiScape^29^ applications of Cytoscape. Mann–Whitney U test was employed to compare the topology features using the SPSS statistical package, version 18.0 (SPSS Inc., Chicago, IL, USA).

The modules were determined in the networks by the MCODE version 1.4.1^30^ plugin of Cytoscape. Human protein–protein interactions were retrieved from HPRD version 9^31^ and imported to the Cytoscape.

### Gene ontology and pathway enrichment analysis

Pathway enrichment analysis was carried out using Cytoscape ClueGO plugin version 2.1.7^32^. In this analysis, Reactome^33^ and KEGG^34^ databases were selected for retrieving the data. In addition, the DAVID database^35^ was applied to collect pathways and biological processes from BioCarta and Gene Ontology (GO), respectively. P-value correction was performed and pathways and GO terms with the adjusted *P*-value ≤ 0.05 were selected.

### Drug target collection

FDA-approved drugs and their target proteins were downloaded from the DrugBank database^36^. Non-human targets were deleted.

### Collection of biochemical features

#### Protein accession and annotation

The UniProt ID and the official protein name for all human proteins were extracted from the UniProtKB/Swiss-Prot of the UniProt database^37^. Using SPSS statistical package, the Chi-square test was employed to compare the biochemical features.

#### Protein family

Human proteins belonging to receptors, G-Couple Protein Receptors (GPCRs), nuclear hormone receptors, enzyme-linked receptors, tyrosine kinase receptors, serine/threonine receptors, ion channels, ligand, and voltage-gated ion channels, transporters, GTPases, ATPases, Phosphatase, and proteases were extracted from protein family of UniProt. In addition, human kinases were collected from the pkinfam data of UniProt.

#### Post-translational modification

Experimentally validated post-translational modifications (PTM) were extracted from the dbPTM database^38^.

#### Enzyme

Enzymes were downloaded from the ENZYME database^39^ and categorized into oxidoreductase, transferase, hydrolase, lyase, isomerase, and ligase classes. Metabolic enzymes were extracted from Metabolic Enzyme Database^40^.

#### Transcription factor and cofactor

The experimentally validated human transcription factors (TFs) list was extracted from the TFCheckpoint database^41^. Transcription cofactors were collected from AnimalTFDB^42^.

#### Epigenetics regulator

The epigenetics regulators including chromatin remodelers, histone modifiers, RNA/DNA modifiers, and scaffold proteins were extracted from EpiFactors^43^.

#### Transcriptional response to small molecules

The list of up or down-regulated genes in the treatment of small molecules identified in the CMAP project was harvested from Enrichr^44^.

#### miRNA target

All human genes identified as strongly validated miRNA targets were retrieved from miRTarBase^22^.

#### Mitochondrial protein

Human proteins located in mitochondria were collected from miToCarta2.0^45^.

#### Mutation

A list of human mutated genes was downloaded from Online Mendelian Inheritance in Man (OMIM)^46^.

#### SNPs-trait association

All SNP-trait associations with *P*-value ≤ 5 × 10^–8^ were obtained from GWAS catalog v2.2.1^47^. SNPs mapped in non-coding and intragenic regions were deleted. Coding genes that were nearest to the SNPs were collected.

### Machine learning

#### State-of-the-art

Current machine learning methods including logistic regression (LR)^14,48,49^, radial basis function (RBF) kernel support vector machine (SVM)^9,12^, generalized linear model (GLM)^50^, and radial basis function network (RBFN)^14^ were utilized to predict potential drug targets. Discriminative features were selected in LR and GLM based on their statistical structures. Sequential Forward Selection (SFS) method was used in SVM and RBFN^51^. The classifiers are briefly described below:

(a) Logistic regression

LR uses the following regression model for the prediction:

$$\log \left( {\frac{p}{1 - p}} \right) = b0 + \sum\limits_{j = 1}^{{N_{f} }} {b_{j} x_{j} } + e$$

where *p* is the detection probability, and *e* denotes the binomial error term. Having fitted the model^52^ by tuning the parameters b_j_ using the input features x_j_ (N_f_ is the number of features), each case with the estimated *p* ≥ 0.5 was classified as the DT, or non-DT otherwise. Upon normalization of the input features, those with low weights could be excluded (a.k.a., feature selection).

(b) Support vector machine

Hyperplanes are mainly used in SVM to separate data points of different classes^51^. The data is transferred to higher dimensions using non-linear mappings (i.e., kernels). We used radial basis function kernels in this study. The method proposed by Wu and Wang was used to tune the radius of the RBF and the soft-margin parameter^53^. Moreover, the SVM classifier was trained using sequential minimal optimization^54^.

(c) Generalized linear model

GLM is a flexible linear regression that involves using a link function to relate the output of the model to the response variable. LR is one of the categories of GLM which employs the logit function i.e., log (p/(1−p)) as the linking function. GLM is suitable for the binomial model. Here, the Poisson linking function was utilized^55,56^.

(d) Radial basis function neural networks

RBF Networks encompass the following layers, the input as the entire features, a hidden layer with a non-linear RBF activation function, and a linear output layer with one node per category or class of data. Each output node calculates the score for the associated class and the class with the highest score is selected for each input sample. The RBF prototypes were estimated using the K-means clustering while the other network parameters were estimated using the Backpropagation algorithm^57^.

#### The mGMDH-AFS algorithm

An mGMDH-AFS algorithm was developed based on inductive neural networks or Group Method of Data Handling (GMDH) that created more accessible models and provide more transparency^58,59^. In biological systems characterized by high dimensionality, it is crucial to perform feature selection to improve classification accuracy^60,61^. The GMDH algorithm was embedded with Particle Swarm Optimization (PSO), a population-based stochastic optimization algorithm^62^, and a Relief-feature-weighting algorithm^63^ to estimate optimal model fitting.

Briefly, categorical data were transferred to interval features using logistic regression^64,65^. The binary encoding was used for each categorical variable, and the logistic regression function parameters were estimated using iterative reweighted least-squares^66^ on the estimation set. It is, indeed, a form of non-linear data normalization. The I-RELIEF algorithm was employed prior to the classification. The weight of the features was iteratively estimated based on their capability to discriminate between neighboring patterns^67^. Moreover, the oversampled^68^ training set was divided into estimation and validation sets to avoid over-fitting^69^.

The GMDH, proposed by Ivakhnenko^70^ was utilized in the current study. In this network, the pairwise interactions of each input feature (a.k.a., neurons) are calculated at each layer. The output of each neuron in the current layer is used as the input to the next layer, and the network is built layer by layer until no improvement is observed in the validation set (i.e., early stopping criterion). The overall structure of the algorithm is provided in Fig. S1.

In this study, the top 10 neurons were selected at each layer. Instead of the traditional polynomial function widely used in the GMDH network, a matrix of nonlinear non-convex functions including exponential, sinusoid, and logarithmic forms was used to model the interaction. Thus, the PSO algorithm was used to estimate the parameters of the model rather than the least-square algorithm. Since the data is highly imbalanced, the Matthews correlation coefficient was used as the fitness function^71^ instead of the traditional RMSE.

The output of the GMDH algorithm is a continuous variable ranging from zero to one. The optimal cut-off was then estimated on the training set using Receiver Operating Characteristic (ROC) curve^72^. The MATLAB code is available online to interested readers: https://github.com/marateb/Drug-Targets-Classification.

#### Validation

The hold-out validation was used to assess the performance of the developed methods. The dataset was randomly split into two independent 70% training and 30% test sets^73^. Also, four-fold cross-validation was employed for further performance assessment to guard against testing hypotheses suggested by the data (Type III errors)^74^. The classifiers were assessed in terms of the performance indices such as sensitivity, specificity, precision, accuracy, and diagnostic odds ratio (DOR) whose definitions are given in Table S1. The Q-Cochran’s test and McNemar’s post-hoc test were used to identify whether the proposed system significantly outperformed the state-of-the-art. The Bonferroni correction was also applied for multiple comparisons and the adjusted *P*-values were then used for interpretation. The random permutation test^75^ was used to compare the performance of the mGMDH-AFS machine with real DT/non-DT classes and ten random sets whose class labels were randomly permuted. MATLAB version 8.6 (The MathWorks Inc., Natick, MA, USA) was used for offline processing.

## Results

### A combination of computational and experimental methods was employed to identify the miRNA profile in DN

**Figure 2:**
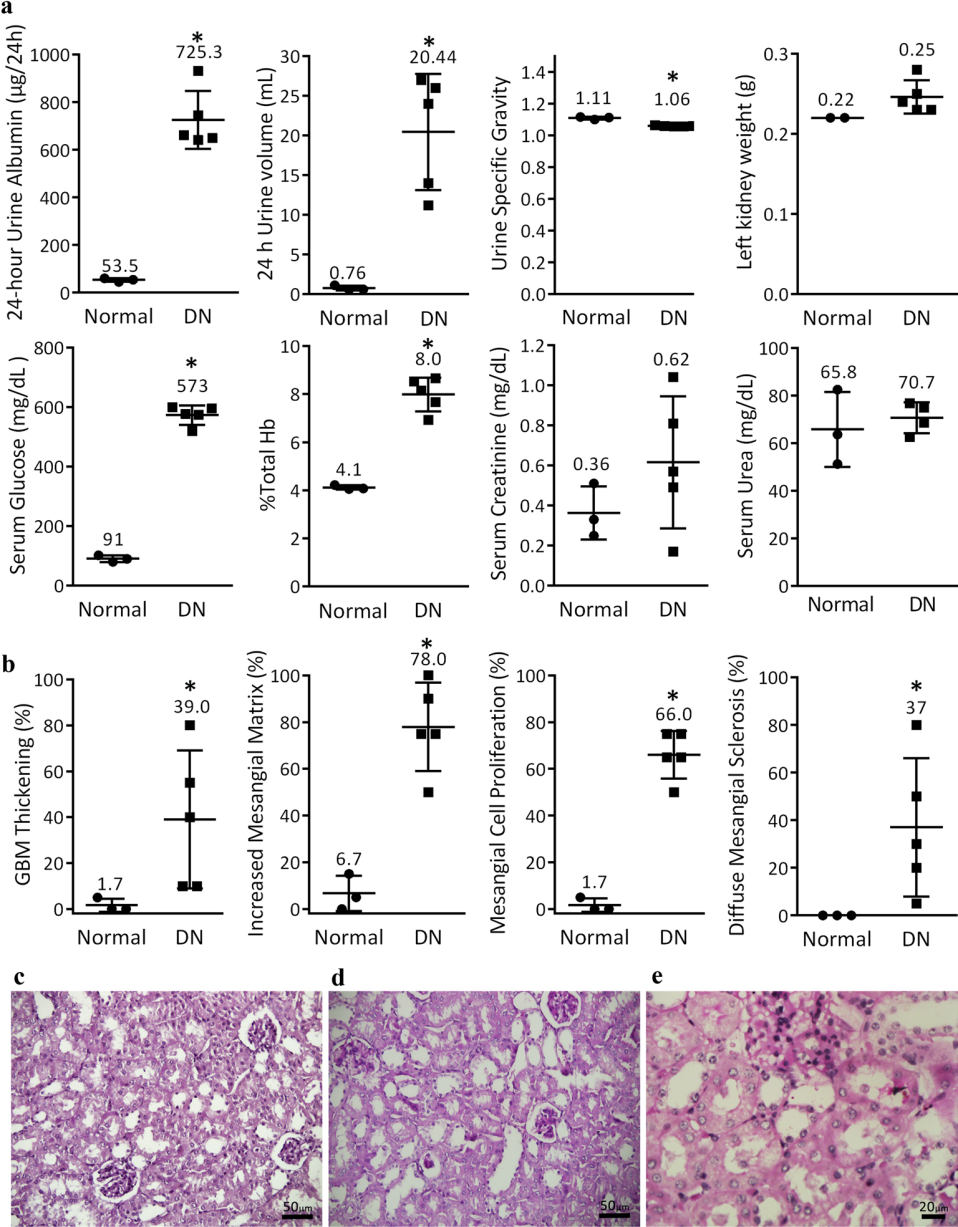
The mouse model of DN was validated with different parameters. A mouse model of DN was established using streptozotocin and validated after 3 months using functional (**a**) and histopathological (**b**) assessments. Representative fields of normal (**c**) and DN (**d**, **e**) kidneys are shown. Data are reported as means ± SD. Asterisks represent *P-*value ≤ 0.05. GBM: glomerular basement membrane.

**Figure 3:**
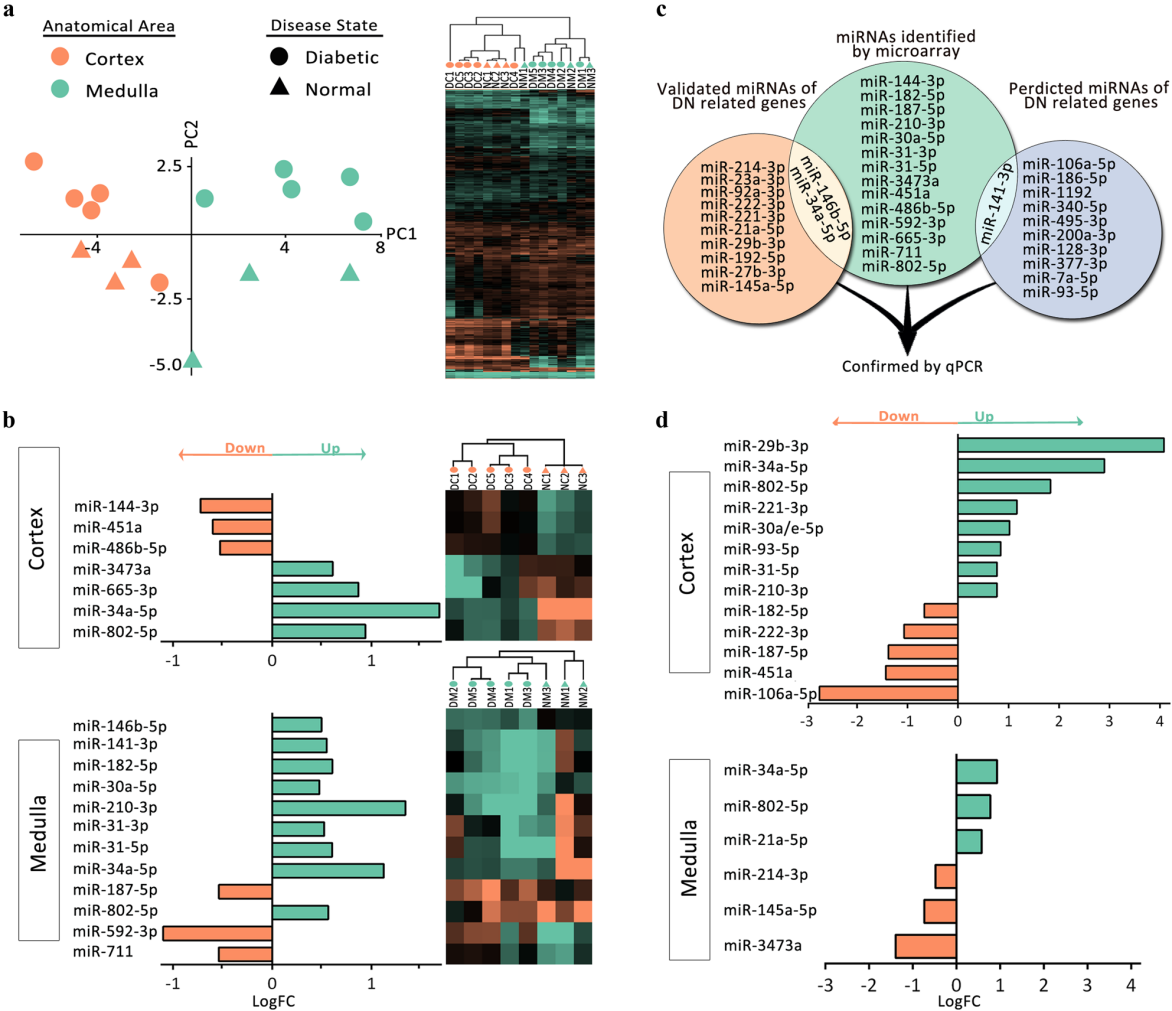
The kidney miRNA profile in DN. To assess microarray data quality in an unsupervised manner, principal component analysis and hierarchical clustering were performed (**a**). In microarray, miRNAs with |logFC|≥ 0.5 in cortex and medulla were determined (**b**). In addition to the miRNAs detected by microarray, some miRNAs which were experimentally shown or predicted to target DN-associated genes were selected (**c**). Among these candidates, 13 and 6 miRNAs were differentially expressed by qPCR in the cortex and medulla, respectively (**d**).

To explore DN pathogenesis, a mouse model of STZ-induced DN was established and after three months was validated using different functional (Fig. 2a) and histopathological (Fig. 2b-e) assessments. For constructing a holistic map of DN, we started with the profile of miRNA related to this disease as these molecules target functionally related genes so each variably expressed miRNA can be a clue to identify a group of related altered genes and functions^5, 76^. miRNA microarray was performed on the cortex and medulla samples, separately, and the quality of microarray data was confirmed using unsupervised hierarchical clustering and PCA (Fig. 3a). We identified 7 and 12 miRNAs with |logFC|≥ 0.5 in cortex and medulla, respectively (Fig. 3b). To propose further miRNAs, a list of genes with a documented role in DN was provided (Table S2). Using TargetScan and miRWalk databases, the predicted and validated miRNAs targeting the DN-associated genes were chosen, respectively (Table S3). Based on the microarray, miRNA prediction, and validation, a total of 37 miRNAs were considered to be potentially related to DN (Fig. 3c). The alternations in the expression of these candidate miRNAs were examined in cortex and medulla samples by quantitative PCR (qPCR). Despite several optimizations, a reliable quantification was not achieved for 8 miRNAs (mmu-miR-711, mmu-miR-592-3p, mmu-miR-186-5p, mmu-miR-495-3p, mmu-miR-1192, mmu-miR-377-3p, mmu-miR-27b-3p, and mmu-miR-146b-5p) due to low or undetectable expression in the kidney or unavoidable technical problems. Among the remaining 29 miRNAs, qPCR data demonstrated the differential expression of 13 and 6 miRNAs in the cortex and medulla, respectively (Fig. 3d).

### A holistic miRNA-target interaction map was constructed, and key functions were inferred

**Figure 4:**
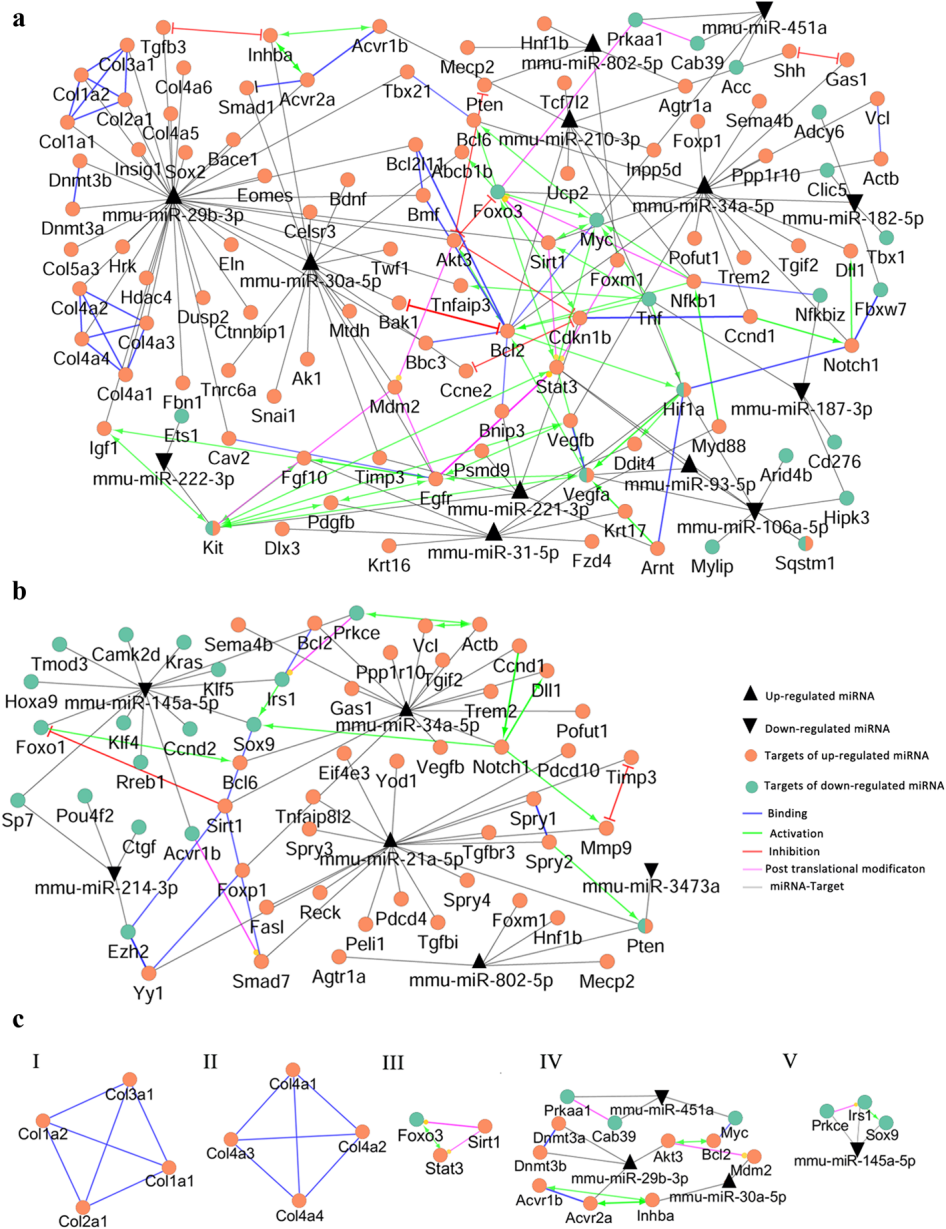
The holistic miRNA-target interaction maps were constructed, and key modules were identified. To investigate the role of differentially expressed miRNAs, the validated targets were identified, and the interaction networks were constructed with the differentially expressed miRNAs and their validated targets in the cortex (**a**) and medulla (**b**). Four modules in the cortex (I-IV) and one in medulla (V) networks were found, which potentially represent key interactions (**c**).

To investigate the role of differentially expressed miRNAs, the validated targets were identified as encompassing 108 and 56 genes for cortex and medulla, respectively (Table S4). Then, the interactions between these genes and their targeting miRNAs were mapped (Fig. 4a, b). As expected for biological networks^77^, both cortex and medulla networks followed a power law distribution. Graph theory measures such as degree, betweenness centrality, and closeness centrality were determined to identify central nodes in the networks. The top genes in terms of these centrality parameters were assumed as central (Table S5). Interestingly, the critical role of the majority of the central nodes such as Hif1a, Vegfa, Sirt1, and Foxo1 has been shown in previous studies^78–81^. Similarly, there are experimental supports for the association of DN and miR-29, miR-34, miR-21, and miR-451, which we identified as central miRNAs^82–85^. This finding is in agreement with the concept that central network nodes drive critical functions^86^.

**Figure 5:**
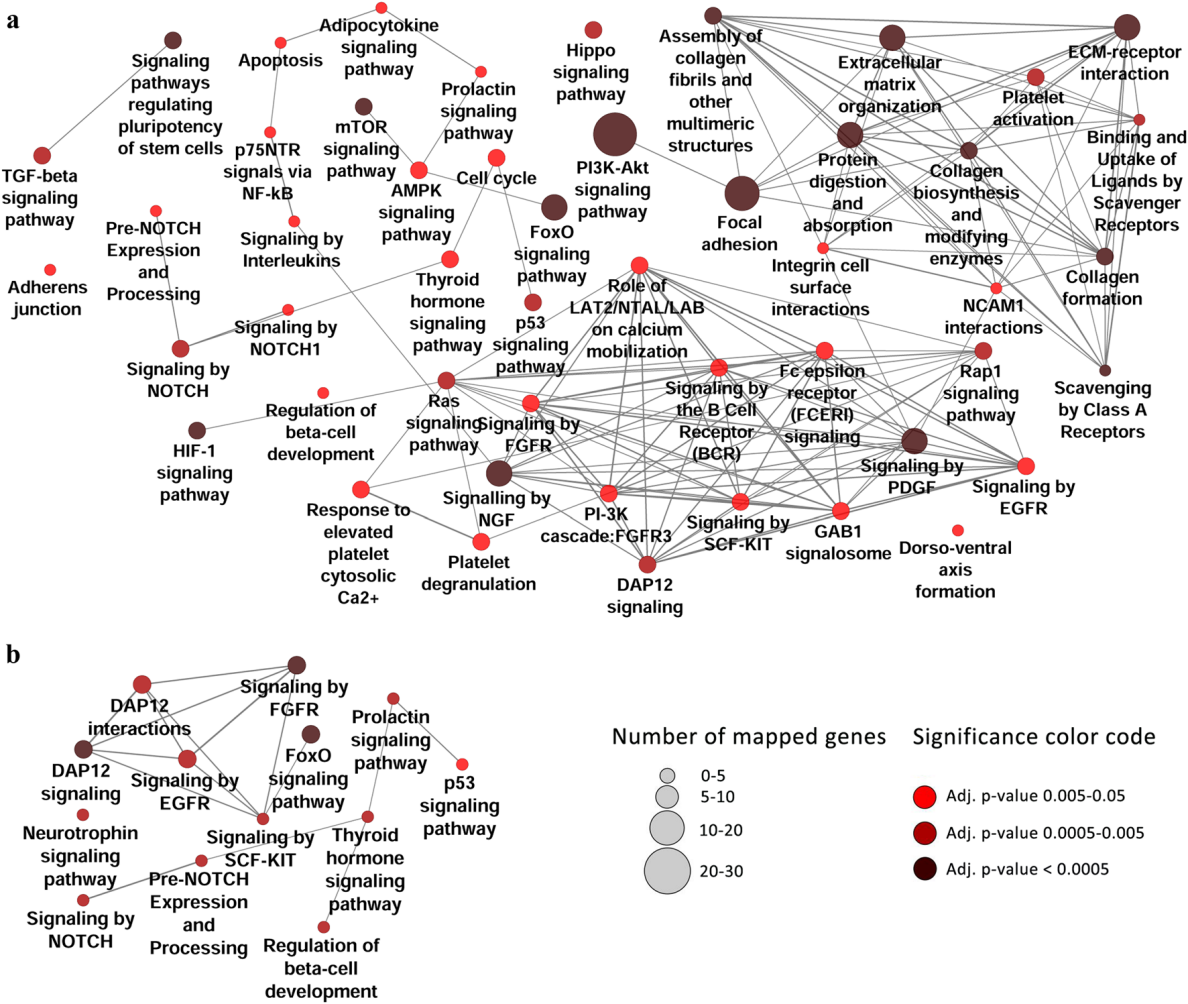
Key signaling pathways associated with DN. Pathway enrichment analysis with miRNA validated targets revealed 40 and 11 inter-connected pathways (adjusted *P* ≤ 0.05) for cortex (**a**) and medulla (**b**), respectively.

To identify key interactions in the cortex and medulla networks, modules were determined as sub-graphs of dense interactions (Fig. 4c). Two modules were related to the extra-cellular matrix aligned with DN histopathology^87,88^. Notably, miR-29, a key player in DN^89,90^ regulates all elements of these two modules. Another module is related to the epigenetic control of Sirt1 on Foxo3 and Stat3, which are shown to be associated with DN pathogenesis^91–93^. Pathway enrichment analysis was performed to identify the signaling pathways associated with the miRNA validated targets. Using KEGG and Reactome databases, forty and eleven inter-connected pathways were enriched for cortex (Fig. 5a) and medulla (Fig. 5b), respectively. Further pathways, as well as GO biological process, were also identified using BioCarta and GO consortium for cortex (Table S6) and medulla (Table S7). The role of most enriched pathways, including TGFB, FGFR, EGFR, Notch, and hypoxia signaling has been shown in DN^94–99^. This analysis was validated by pathway enrichment analysis for ten random gene lists of similar sizes which yielded no or a few unrelated signaling pathways (data not shown).

### A novel high-performance machine learning method was developed to predict potential drug targets

**Figure 6:**
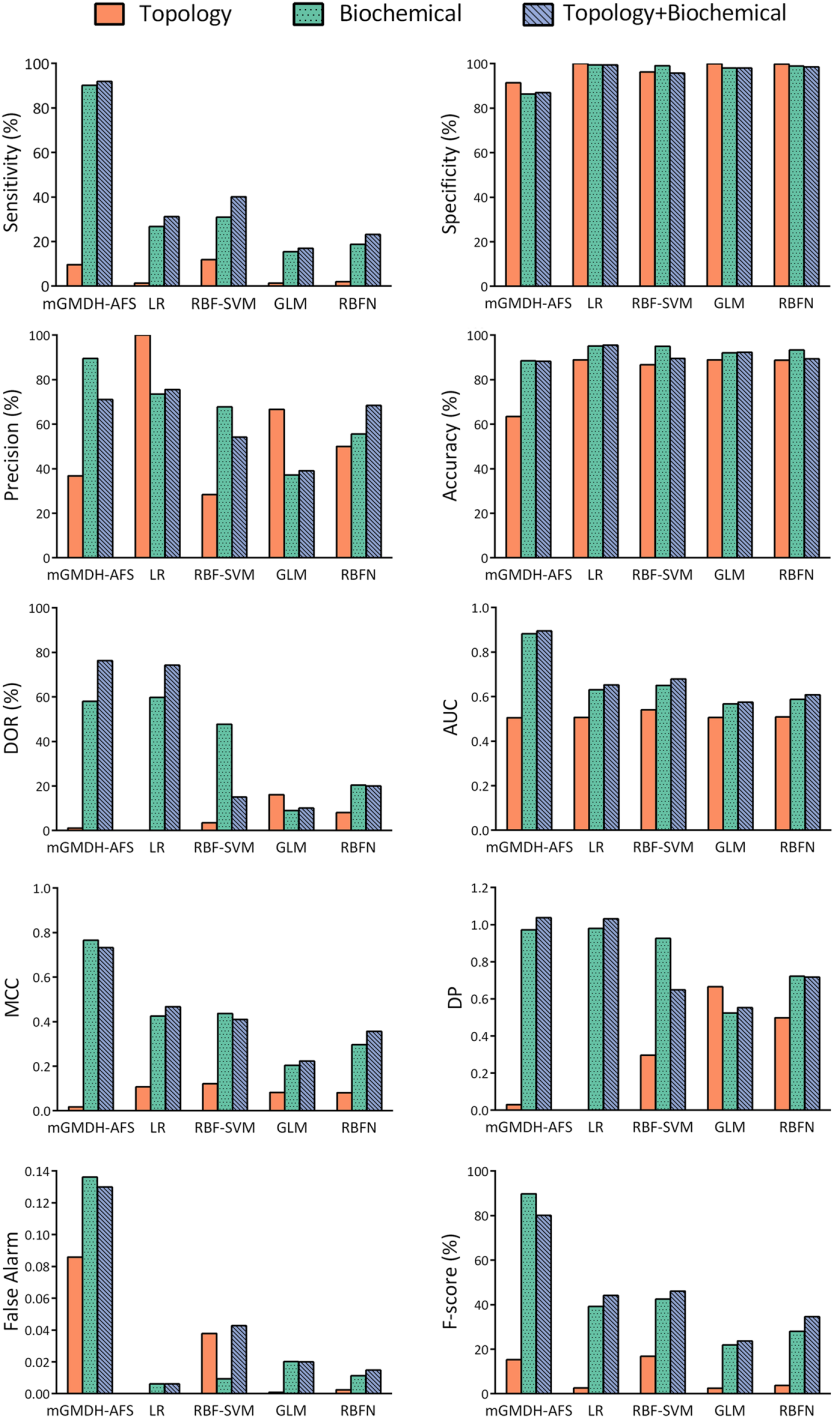
The novel machine could appropriately classify human proteins as drug targets or non-drug targets. The performance of the proposed mGMDH-AFS machine for drug target prediction based on biochemical or topology + biochemical features was acceptable and significantly superior to the examined standard machines including logistic regression (LG), radial basis function kernel support vector machine (RBF-SVM), generalized linear model (GLM), and radial basis function network (RBFN) as revealed by the hold-out validation. The proposed method significantly outperformed the state-of-the-art models (adjusted *P* ≤ 0.05). AUC: area under the receiver operating characteristic (ROC) curve; DOR: diagnosis odds ratio; MCC: Matthews correlation coefficient; DP: discriminant power.

One of the main objectives of this research was to translate the findings on DN molecular pathogenesis into clinical applications. Thus, a machine learning approach was developed to predict which role player molecules could be suitable therapeutic targets. We followed the hypothesis that FDA-approved drug targets have unique properties compared to other proteins and these characteristics can be used to propose novel drug targets. Sixty-five biochemical features (Table S8) were determined which were harvested from several databases, for all human proteins (#20,132). Moreover, 23 network topology features (Table S9) were determined for the proteins in the human interactome obtained from HPRD (#9226). Based on DrugBank, 1443 proteins were then determined to be the targets of at least one FDA-approved drug. These proteins were considered as the DT group while the remaining 18,689 proteins were assigned to the non-DT class. Although the frequency or median of most biochemical (Fig. S2) and topology (Fig. S3) features are statistically different between DT and non-DT proteins, extensive distribution overlaps can be observed between these two protein classes. To assess the predictive value of these features for discrimination between DT and non-DT, different standard machine learning algorithms, namely logistic regression (LG), radial basis function kernel support vector machine (RBF-SVM), generalized linear model (GLM), and radial basis function network (RBFN) were utilized. The hold-out method measurements showed that the performance of none of the exploited methods was satisfying, neither with biochemical nor topology features (Fig. 6).

The failure of classical machines could be due to the unique nature of the data such as the considerable imbalanced size of the groups, significant overlap in the distribution of features in the two protein classes, and high dimensionality. The mentioned limitations were addressed by developing mGMDH-AFS, a high-performance machine learning method that considers high-level feature interactions. The performance of this novel tool was acceptable and significantly superior to the other standard machines, as revealed by hold-out validation (Fig. 6). The prediction power of mGMDH-AFS was further evaluated by fourfold cross-validation (Fig. S4). While topology features alone were not predictive for discrimination between DT and non-DT classes, biochemical features and the combination of biochemical and topology features were informative for the classification. The core topology features selected by mGMDH-AFS were included network degree, betweenness, and closeness. Also, being an enzyme, a receptor, an ion channel, or having post-translational modifications (PTM) were amongst the main biochemical features. To further assess the functionality of mGMDH-AFS, all human proteins were randomly labeled as 1 and 0 with the ratio of 1/0 the same as DT/non-DT classes. This procedure was repeated ten times to generate ten sets of randomly allocated proteins. As expected, the machine performance was significantly better with the real set compared to these random sets, indicating a reliable classification (Fig. S5).

**Figure 7:**
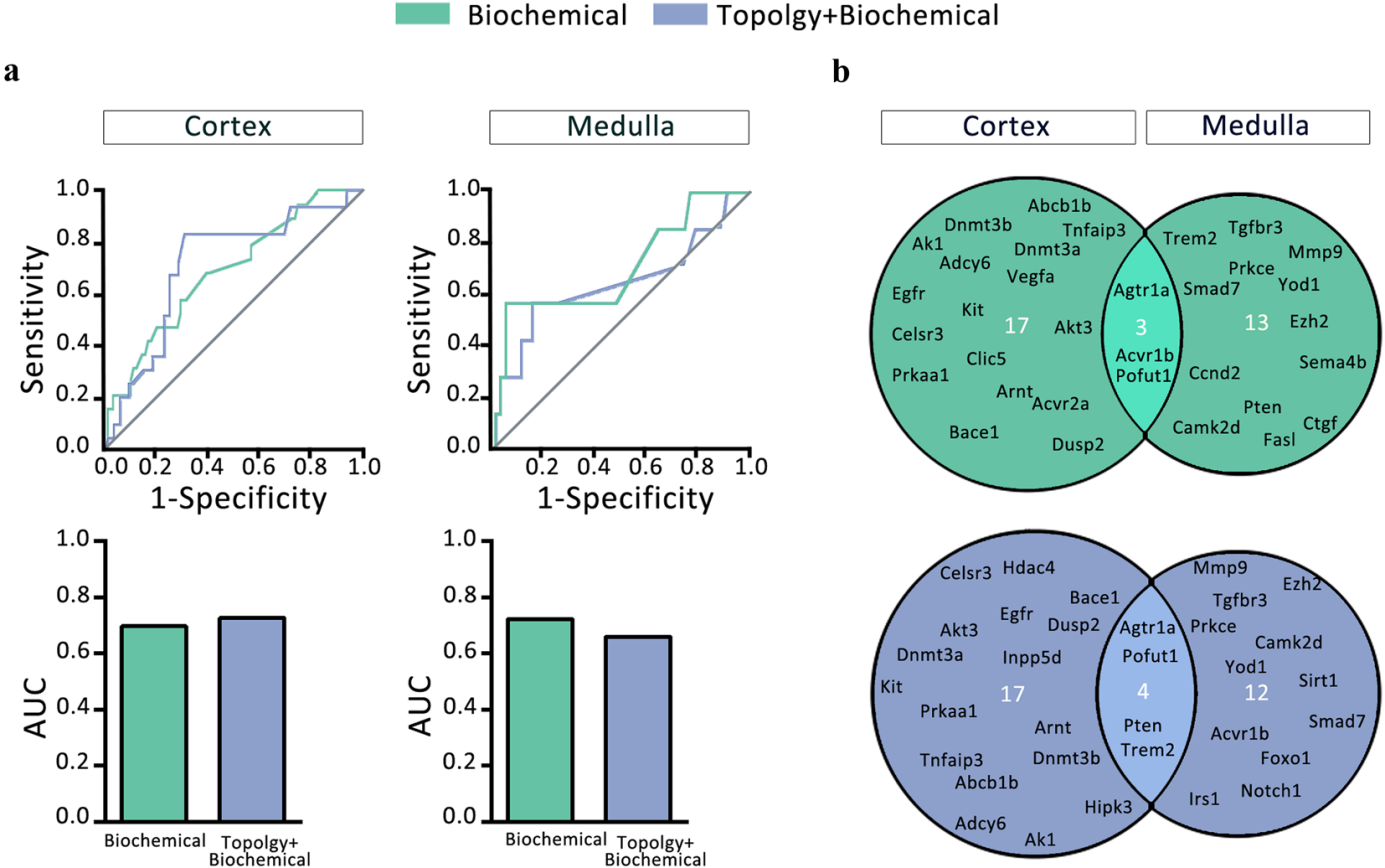
The developed machine learning algorithm proposed novel therapeutic targets for DN. The performance of the mGMDH-AFS classifier to predict drug targets in the constructed networks of cortex and medulla was assessed by ROC curve analysis (**a**). The top predicted targets in cortex and medulla networks are shown either with biochemical or topology + biochemical features (**b**).

After validating the mGMDH-AFS performance for all human proteins, this classifier was applied to predict potential DT in the constructed cortex and medulla networks. The algorithm calculated the probability of being DT for each protein in the networks. Using DrugBank to identify currently approved targets in these networks, an ROC curve analysis was carried out to evaluate machine performance (Fig. 7a). The top predicted drug targets for both cortex and medulla are Agtr1a, Egfr, Kit, Celsr3, Clic5, Tgfbr3, Acvr1b (Fig. 7b). Interestingly, the machine predicted Angiotensin II receptor, a well-known current drug target in DN, as the best candidate in the medulla and the 3rd best one in the cortex, supporting the validity of the developed approach. Some experimental supports were also found for other proposed targets such as EGF receptor and protein kinase C isoforms in previous studies on DN^100, 101^.

## Discussion

Despite considerable efforts, diabetic patients progress to end-stage renal disease at alarming proportions, necessitating further investigations to design more efficient therapeutic approaches^102^. Numerous studies have shown the role of individual elements in the pathogenesis of DN but systematic evaluations have rarely been attempted. In this study, miRNA-targets interaction maps were established in DN using system biology approaches. The central nodes, modules, and critical signaling pathways were determined. To translate the research findings to clinical applications, an innovative high-performance classifier was also developed to predict novel therapeutic targets in DN.

The selection of miRNAs as the starting point for investigating DN molecular pathogenesis was based on the concept that they target functionally related genes. Indeed, each variably expressed miRNA can be a clue to identify a group of related altered functions^5,76^. Microarray profiling and bioinformatics predictions were employed in parallel to identify some miRNAs for further validation with qPCR. Among the identified DE miRNAs, the roles of miR-34a, miR-29b, miR-21a, miR145a, and miR-451a have been extensively shown in DN^83,84,89,103,104^, indicating the validity of our approach. However, to the best of our knowledge, miR-802-5p, 182-5p, 210-3p, 31-5p, and 106a-5p are for the first time attributed to DN in the current study.

One of the advantages of the present study is separate miRNA profiling for the cortex and medulla. Remarkably, the PCA plot demonstrated that the effect of anatomical area on miRNA expression was higher than the impact of the disease state. Although miR-802-5p and miR-34a are overexpressed in both cortex and medulla of DN mice, the other identified miRNAs are differential in either cortex or medulla. This finding is in line with recent studies revealing diverse mRNA and miRNA expression profiles for different anatomical parts of the normal kidney^105,106^.

To provide evidence about the role of the identified miRNAs, their targets were collected and miRNA-target interaction networks were constructed. The constructed networks allowed a global systematic view towards the collaborations between miRNAs and their interactions with corresponding targets. Considering the complexity of biological interaction networks, it is critical to identify the elements with the highest influence on the outcome. Therefore, we performed topology analysis to determine the central nodes in the networks, as it has been previously shown that they drive key signaling pathways^86^. Interestingly, the in vivo knockdown of miR-29, one of the central miRNAs in the cortex network, has been shown to halt the progression of DN^82^. Notably, in the cortex network, this miRNA regulates two modules composed of extracellular matrix (ECM) elements. The identification of two modules associated with ECM in the cortex network is aligned with the histopathologic finding of fibrosis in the cortex.

The role of DE miRNAs was further explored by performing pathway enrichment analysis to determine the signaling pathways associated with miRNA targets. Most of the enriched pathways, including TGFB, EGFR, and Notch signaling pathways were experimentally shown to be associated with DN^107–109^. Using this analysis, we could also predict novel pathways whose roles in DN remain to be confirmed in future studies. NGF signaling pathway which is related to diabetic neuropathy was amongst the enriched pathways^110^. Similarly, the platelet degranulation pathway was detected as a potential role player in DN. Previous studies have demonstrated the role of this pathway in some pro-fibrotic disorders such as idiopathic pulmonary fibrosis and myelofibrosis^111,112^.

The findings of this research were translated to clinical applications. It is hypothesized that the current FDA-approved drugs affect proteins with distinctive properties and these features can potentially be used to introduce novel drugs. Although some studies have shown that current DTs have unique features^8,113^, the construction of classifiers has remained a major challenge due to the overlap of feature distributions. To distinguish these two protein classes, some previous investigators have employed common machine learning approaches including LR, SVM, GLM, and RBFN^9,14,48,50^. In this study, however, these methods did not lead to satisfying results. This discrepancy could be assigned to the fact that these studies used equal size DT and non-DT classes to increase machine performance, or assessed the outputs with limited indices.

To address the limitations of current classifiers, a high-performance machine learning method was developed. Unlike other methods, we considered the original highly imbalanced datasets for classification. However, learning from the imbalanced data is challenging^114^. In this algorithm, a cost function other than the traditional mean-square-error metrics was utilized to avoid learning bias toward the majority class. The fitness function of the Matthews correlation coefficient was used whose robustness in imbalanced datasets has been proven^71,115^.

In addition to the initial feature selection using the Relief algorithm, different interactions of the selected input features were considered during GMDH deep learning procedure. Indeed, biological systems are very complex and the nonlinear interactions of the non-redundant features could improve the performance of their classification systems^116^. Based on different assessments, the performance of mGMDH-AFS is superior to that of the state-of-the-art approaches, suggesting it as a promising approach for therapeutic target prediction in complex disorders.

After several validation steps, the developed machine learning algorithm was employed to analyze the cortex and medulla networks. It provided well-balanced diagnostic accuracy rates and resulted in a list of novel promising therapeutic targets for DN, which can be assessed in upcoming investigations. Among the top-ranked candidates in the cortex or medulla are Agtr1a, Egfr, Clic5, and Prkce. Interestingly, Agtr1a is the target of angiotensin receptor blockers which are currently in the market for DN treatment^117^. It has been also shown that the inhibition of Egfr or Prkc isoforms by small molecules can prevent the progression of DN^100,101^.

In conclusion, a combination of experimental and computational methods was exploited to generate a holistic map of DN and introduce novel therapeutic targets. The limitation of this work is the restriction of experimental data to miRNA profiling. As a future perspective, we plan to integrate other omics layers into the constructed networks to achieve more accurate insights. Notably, the proposed approach for drug target prediction could also be employed for other complex disorders as well.

## Supplementary Information

Supplementary Tables.

Supplementary Figures.
