# Supplementary material for: Systems biology and machine learning approaches identify drug targets in diabetic nephropathy
Source: Sci Rep. 2021 Dec 6;11:23452. doi: 10.1038/s41598-021-02282-3 (PMC8648918; doi:10.1038/s41598-021-02282-3)
Supplement: Supplementary file 2 — Supplementary Figures. [file 41598_2021_2282_MOESM2_ESM.docx]

**Systems Biology and Machine Learning Approaches Identify Drug Targets in Diabetic Nephropathy**

Maryam Abedi ^1^, Hamid Reza Marateb ^2, 1, 3^, Mohammad Reza Mohebian ^4^, Seyed Hamid Aghaee-Bakhtiari ^5, 6^, Seyed Mahdi Nassiri ^7^, Yousof Gheisari ^1, 8*^

1. Regenerative Medicine Research Center, Isfahan University of Medical Sciences, Isfahan, Iran
2. Biomedical Engineering Department, Engineering Faculty, University of Isfahan, Isfahan, Iran
3. Department of Automatic Control, Biomedical Engineering Research Center, Universitat Politècnica de Catalunya, BarcelonaTech (UPC), Barcelona, Spain
4. Department of Electrical and Computer Engineering, University of Saskatchewan, Saskatoon, Canada
5. Biotechnology Research Center, Mashhad University of Medical Sciences, Mashhad, Iran
6. Department of Medical Biotechnology, Mashhad University of Medical Sciences, Mashhad, Iran
7. Department of Clinical Pathology, Faculty of Veterinary Medicine, University of Tehran, Tehran, Iran
8. Department of Genetics and Molecular Biology, Isfahan University of Medical Sciences, Isfahan, Iran

* Corresponding Author:

Yousof Gheisari MD, PhD. Regenerative Medicine Research Center, Isfahan University of Medical Sciences, Isfahan, 8174673461, Iran

Tel/Fax: +98-3136687087. Email: ygheisari@med.mui.ac.ir

Contents

Figure S1 3

Figure S2 4

Figure S3 5

Figure S4 6

Figure S5 7


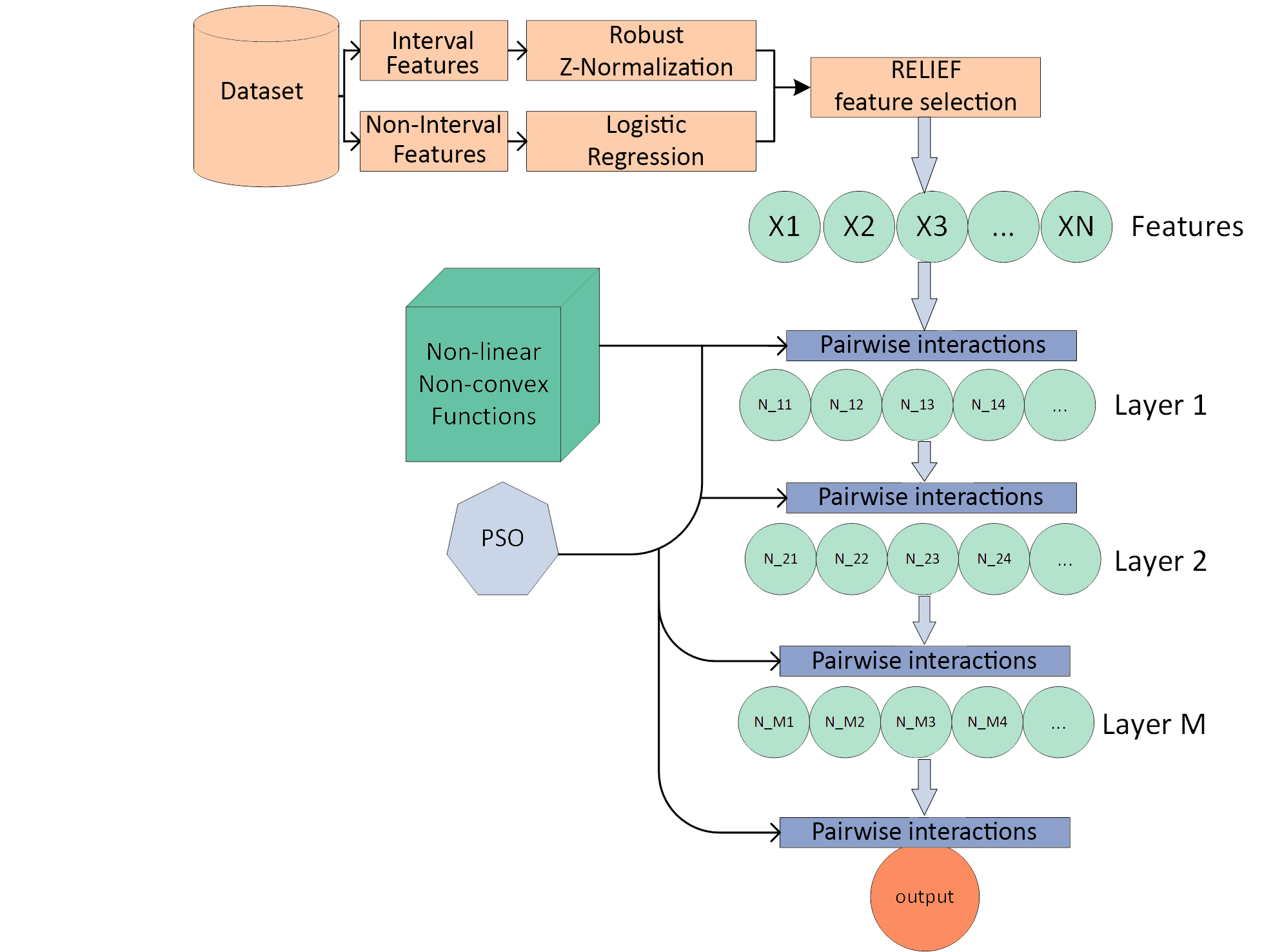


Figure S1**-** The overall structure of the proposed algorithm.

**
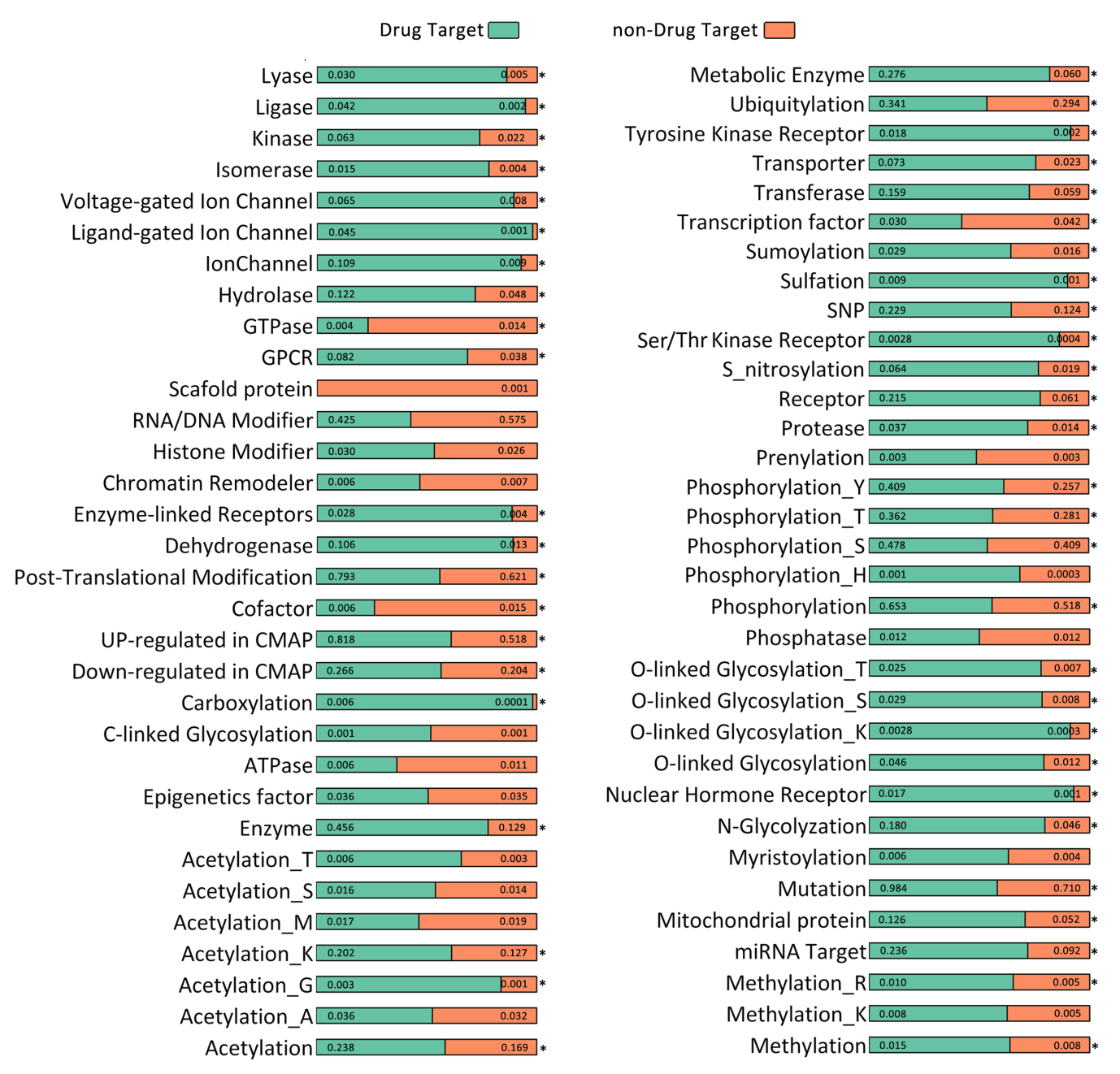
**

Figure S2**- All human proteins were annotated for 65 biochemical features.** In each bar, the left and right numbers indicate the relative frequencies of proteins that are positive for the feature among all drug target and non-drug target proteins, respectively. The features whose relative frequencies are statistically different (*P-*value ≤ 0.05) between these two protein classes are marked with asterisks**.**

**
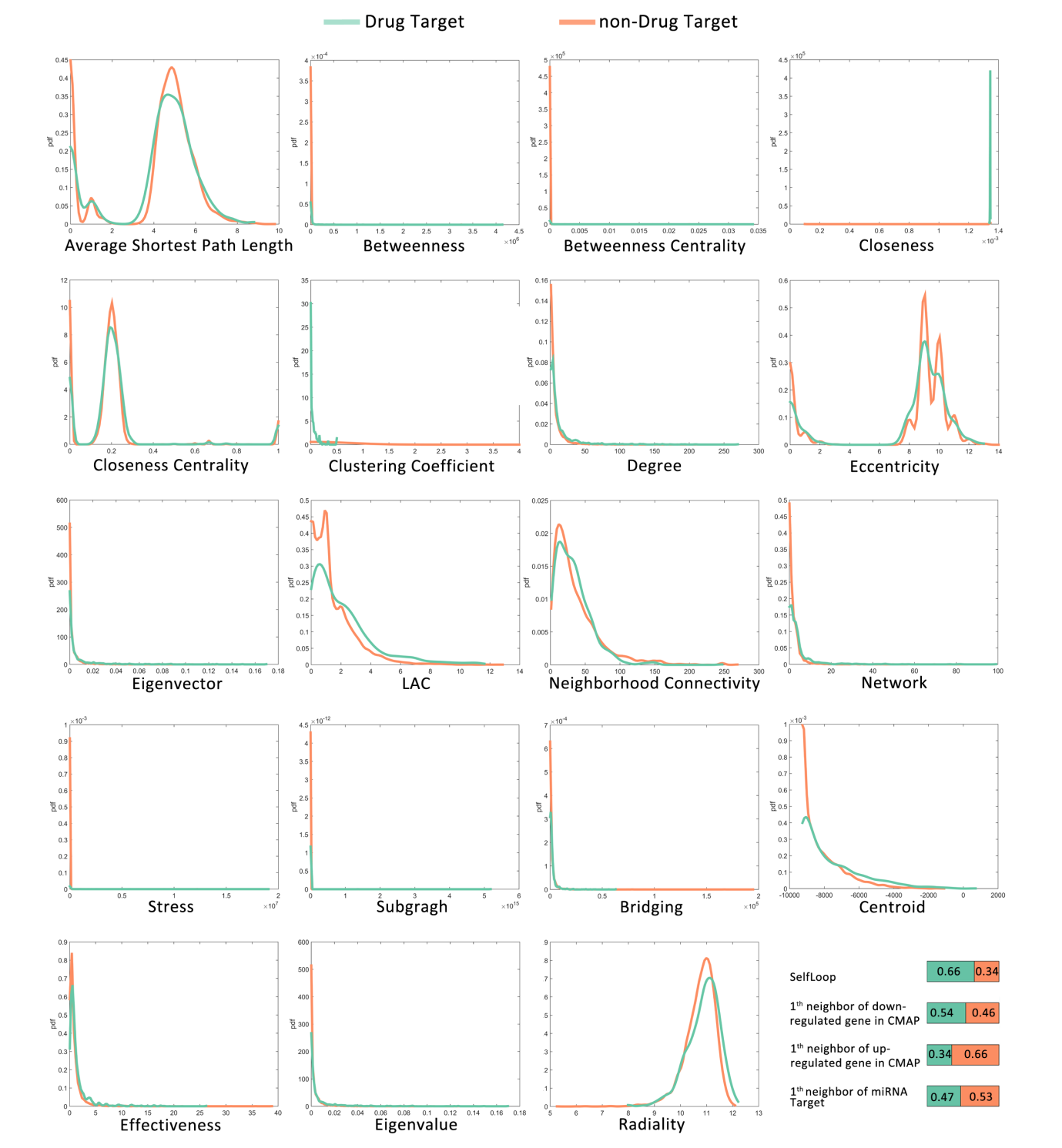
**

Figure S3**- Different topology features were determined for the proteins in the human interactome.** Twenty-three network topology features were determined for 9226 proteins in the human interactome obtained from HPRD. These features show extensive distribution overlaps between drug target and non-drug target proteins. However, the median of all features except the closeness and closeness centrality are statistically different between these two protein classes (*P*-value ≤ 0.05). LAC: Local average connectivity-based method.

**
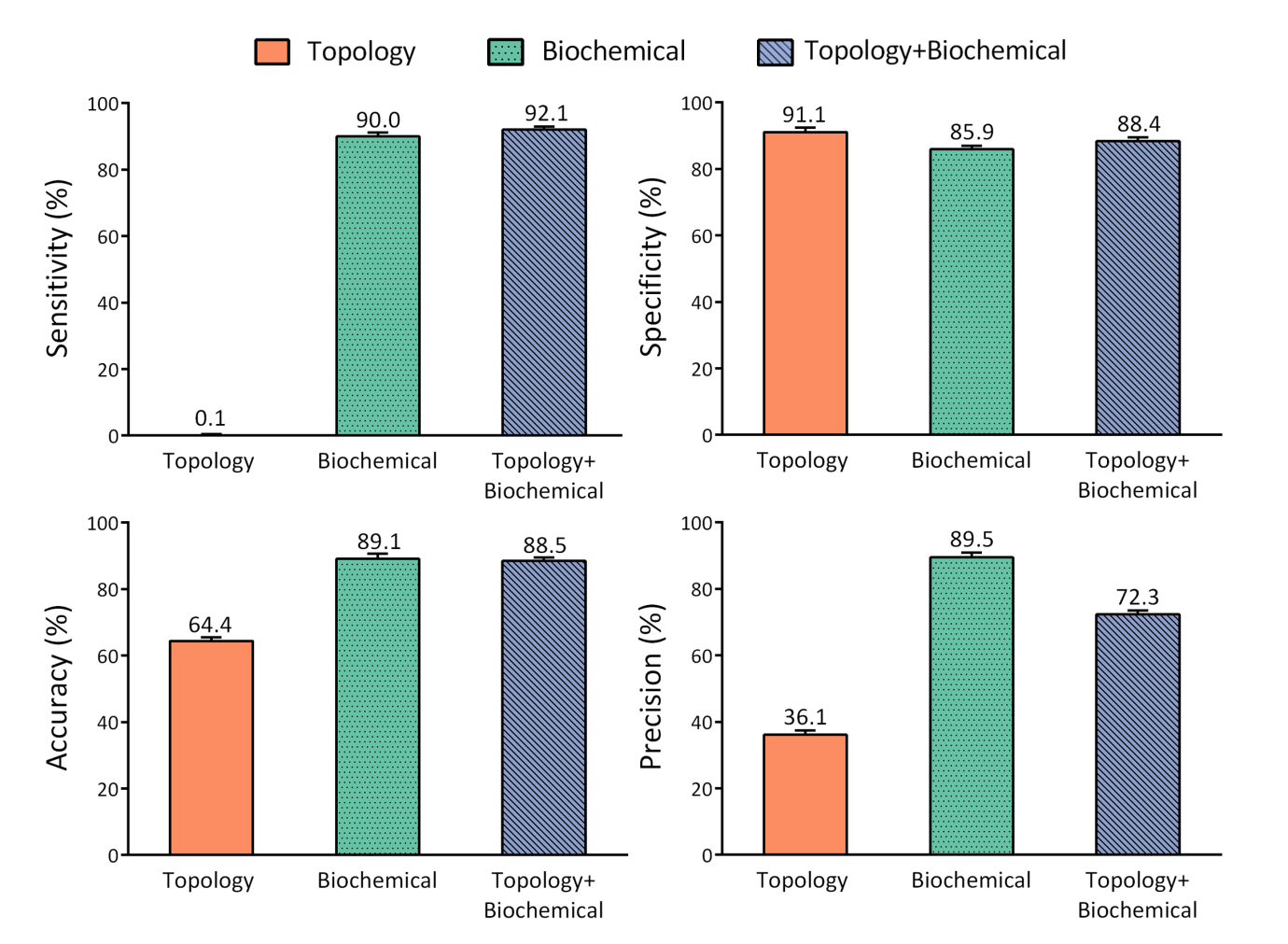
**

Figure S4**-** **The prediction power of mGMDH-AFS was further evaluated by 4-fold cross-validation.** To guard against testing hypotheses suggested by the data, the functionality of the mGMDH-AFS machine for drug target prediction was also assessed by 4-fold cross-validation. The error bars represent the standard deviation.

**
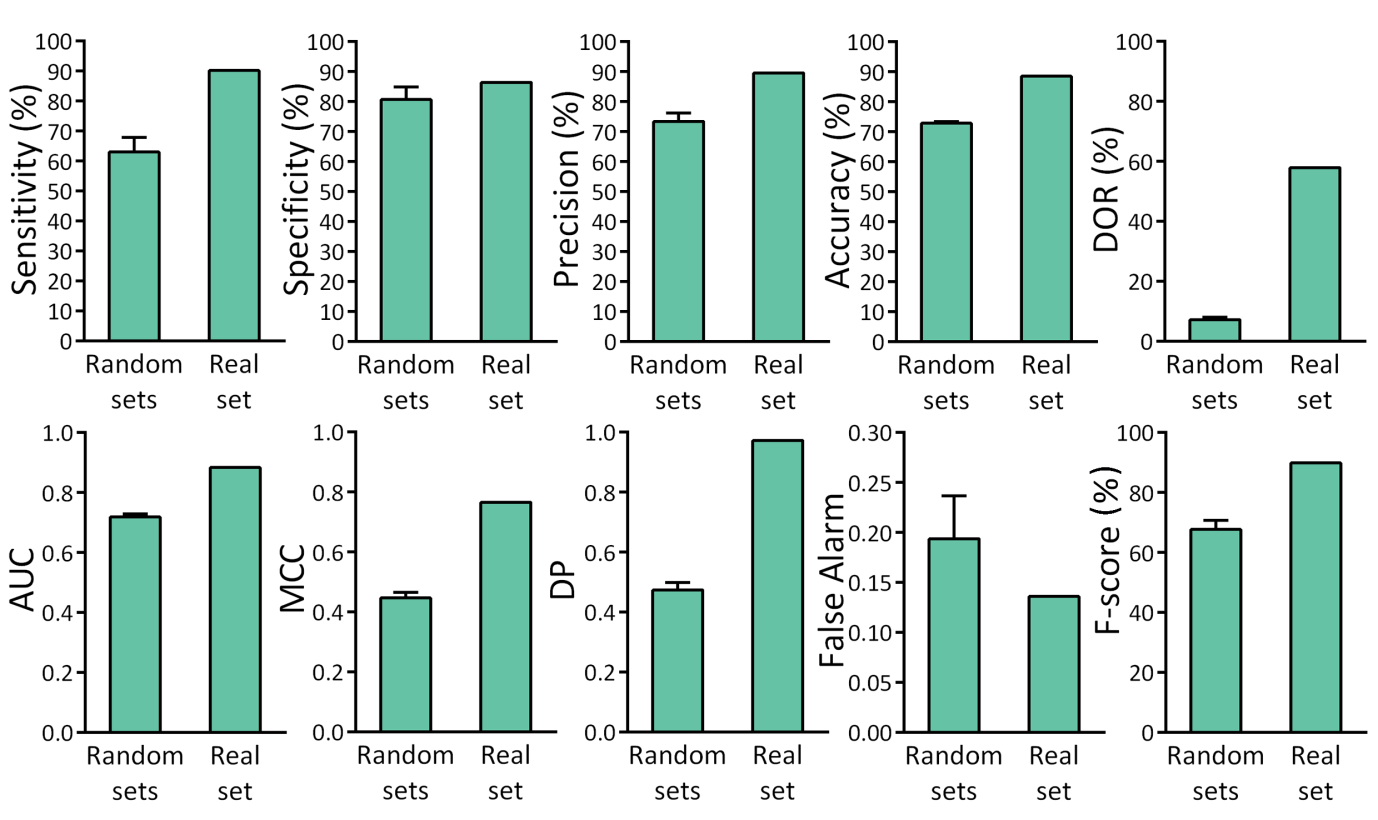
**

Figure S5**-** **The performance of mGMDH-AFS was further assessed with random sets.** As 1443 of all human proteins are known to be targets of FDA-approved drugs, ten random sets of human proteins were generated. In each of which, 1443 proteins were arbitrarily assigned as positive hits. The random permutation test identified that the proposed model was significantly better than the NULL model (i.e. 10 random permitted models) (*P* ≤ 0.05). The biochemical features were used for this assessment.
